# High-Entropy Layered Oxide Cathode Enabling High-Rate for Solid-State Sodium-Ion Batteries

**DOI:** 10.1007/s40820-023-01232-0

**Published:** 2023-11-09

**Authors:** Tianxun Cai, Mingzhi Cai, Jinxiao Mu, Siwei Zhao, Hui Bi, Wei Zhao, Wujie Dong, Fuqiang Huang

**Affiliations:** 1 State Key Laboratory of High Performance Ceramics and Superfine Microstructure, Shanghai Institute of Ceramics, Chinese Academy of Sciences, Shanghai, 200050 People’s Republic of China; 2 State Key Laboratory of Rare Earth Materials Chemistry and Applications, College of Chemistry and Molecular Engineering, Peking University, Beijing, 100871 People’s Republic of China; 3 Center of Materials Science and Optoelectronics Engineering, University of Chinese Academy of Sciences, Beijing, 100049 People’s Republic of China; 4 Zhongke Institute of Strategic Emerging Materials, Yixing, 214213 Jiangsu People’s Republic of China

**Keywords:** High-entropy, High-rate performance, Li–TM interaction, Air stability, O3 layered oxide cathode

## Abstract

Na-ion O3-type layered oxides are prospective cathodes for Na-ion batteries due to high energy density and low-cost. Nevertheless, such cathodes usually suffer from phase transitions, sluggish kinetics and air instability, making it difficult to achieve high performance solid-state sodium-ion batteries. Herein, the high-entropy design and Li doping strategy alleviate lattice stress and enhance ionic conductivity, achieving high-rate performance, air stability and electrochemically thermal stability for Na_0.95_Li_0.06_Ni_0.25_Cu_0.05_Fe_0.15_Mn_0.49_O_2_. This cathode delivers a high reversible capacity (141 mAh g^−1^ at 0.2C), excellent rate capability (111 mAh g^−1^ at 8C, 85 mAh g^−1^ even at 20C), and long-term stability (over 85% capacity retention after 1000 cycles), which is attributed to a rapid and reversible O3–P3 phase transition in regions of low voltage and suppresses phase transition. Moreover, the compound remains unchanged over seven days and keeps thermal stability until 279 ℃. Remarkably, the polymer solid-state sodium battery assembled by this cathode provides a capacity of 92 mAh g^−1^ at 5C and keeps retention of 96% after 400 cycles. This strategy inspires more rational designs and could be applied to a series of O3 cathodes to improve the performance of solid-state Na-ion batteries.

**Supplementary Information:**

The online version contains supplementary material available at 10.1007/s40820-023-01232-0.

## Highlights

- High-entropy oxides O3-Na_0.95_Li_0.06_Ni_0.25_Cu_0.05_Fe_0.15_Mn_0.49_O_2_ cathode constructed by compatible radius and different Fermi level ions was designed for solid-state Na-ion batteries.
- Na_0.95_Li_0.06_Ni_0.25_Cu_0.05_Fe_0.15_Mn_0.49_O_2_ cathode exhibits high-rate performance, air stability and electrochemically thermal stability.
- A series of characterizations were performed to explore energy storage mechanism of Na_0.95_Li_0.06_Ni_0.25_Cu_0.05_Fe_0.15_Mn_0.49_O_2_.

**Supplementary Information:**

The online version contains supplementary material available at 10.1007/s40820-023-01232-0.

## Graphical abstract

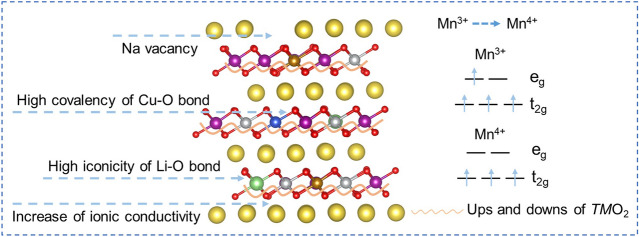

## Introduction

Sodium-ion batteries (SIBs) have attracted huge attention as a prospective alternative to lithium-ion batteries (LIBs), particularly in large-scale energy storage because of the abundance and low price of Na resources, as well as similar chemical properties to the commercial LIB [1–3]. The Na-ion layered oxide materials, Na_*x*_*TM*O_2_ have considerable potential as cathode materials for NIBs owing to the notable benefits including high energy density, facile production and cost-effectiveness. Designing cathode materials possessing high Na content and rapid diffusion kinetics is a crucial aspect in achieving commercial application. Compared with P2-type cathodes, O3-type materials possess higher sodium content, rendering them desirable to couple with sodium-free anode [4, 5]. Nevertheless, the potential for their actual application is constrained by some significant limitations, namely, the complex phase transitions, the sluggish Na^+^ diffusion kinetics and the inherent sensitivity to air. Layered O3-type Na_*x*_*TM*O_2_ displays more complex phase transitions in comparison with its Li analogs due to larger Na^+^ ions associated with charge ordering and the ordering arrangement between Na^+^ and vacancies [5, 6]. The slower sodium-ion kinetics of O3-type material is an inherent characteristic due to its narrower spacing between sodium interlayers. In O3 framework, the migration of Na^+^ between octahedral sites must pass via a tetrahedral site, resulting in a high energy barrier owing to the difference in size between the large Na-ion and confined tetrahedral voids [7, 8]. On the other hand, in the presence of air, O3 materials experience the generation of active Na on the surface. This process is accompanied by the structure aberration and the oxidation of transition metal ions within the bulk. The segregated Na undergoes a fast reaction with H_2_O/CO_2_ in ambient air. This reaction leads to the creation of NaOH or Na_2_CO_3_ on the surface of active materials, hence causing the deteriorated battery performance [7, 9, 10]. Therefore, searching for a strategy to resolve these unfavorable factors is critical for realizing high performance solid-state Na-ion batteries.

Some investigations have demonstrated that the appropriate element substitutions (such as Ti^4+^, Mg^2+^, Cu^2+^, Al^3+^, Sn^4+^, Zn^2+^, etc.) considerably suppress the irreversible phase transition and improve Na^+^ diffusion coefficient of O3-type oxides [11–15]. However, in order to achieve better performance, the O3-type material is still needed to be optimized. High-entropy oxides (HEOs) have gained significant attention in the realm of electrochemical energy storage, owing to its distinctive structure and exceptional performance [16–20]. HEOs are regarded as a stable solid solution phase consisting of five or more elements in equimolar or nearly equimolar ratios (each element content is within the range of 5–35%) [21, 22]. Zhao et al. successfully prepared a high-entropy O3-type layered oxide NaNi_0.12_Cu_0.12_Mg_0.12_Fe_0.15_Co_0.15_Mn_0.1_Ti_0.1_Sn_0.1_Sb_0.04_O_2_ cathode. The HEO cathode exhibited remarkable cycling stability, with a capacity retention of 83% after 500 cycles and enhanced rate performance, retaining 80% capacity at 5C. Furthermore, the researchers also revealed that the high-entropy structure can prolong the phase transition, which is beneficial to improve the cycling stability of cathode materials [20].

**Fig. 1:**
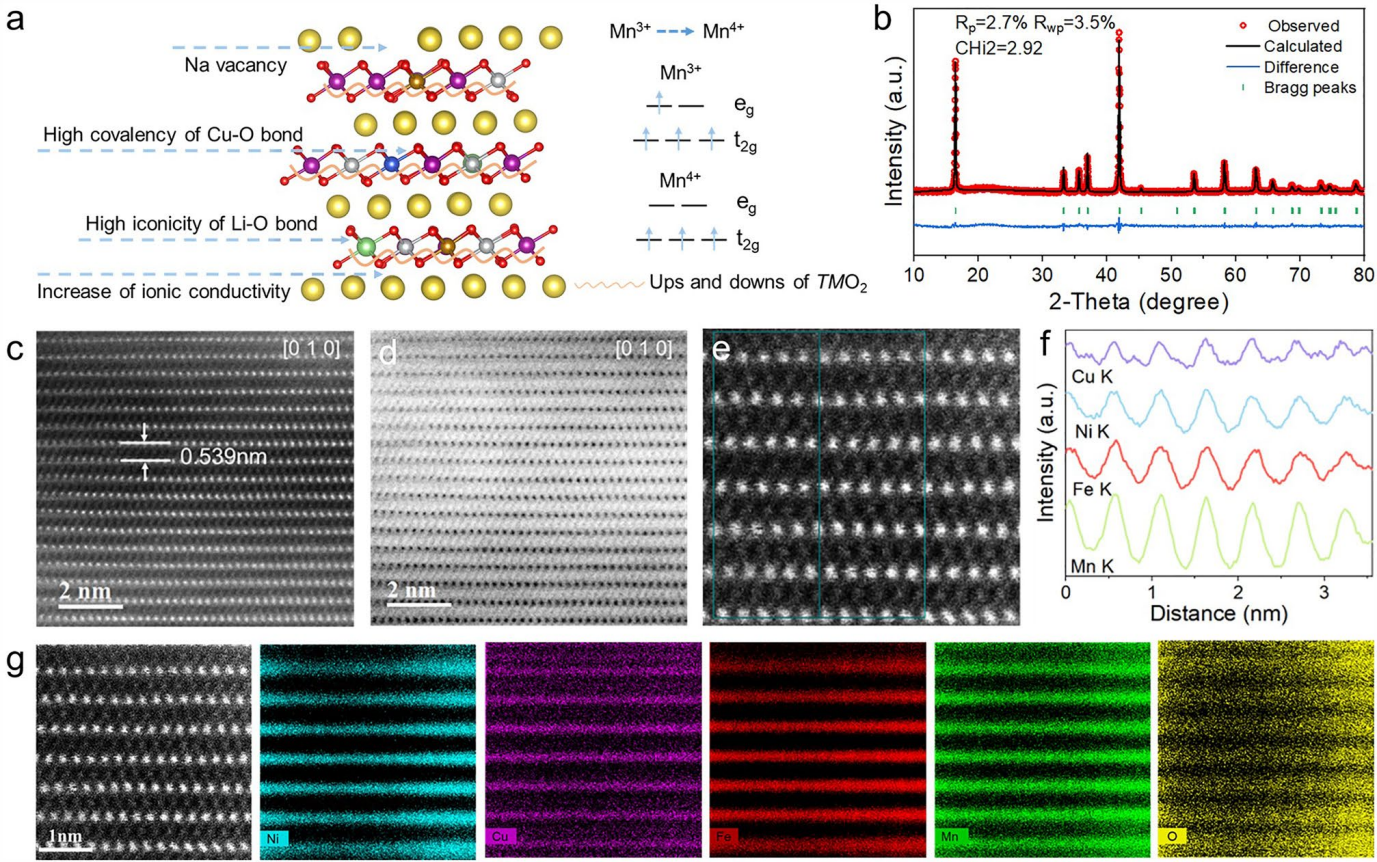
Structure characterization of Na_0.95_LNCFM materials. **a** Schematic of O3-type structure design. **b** XRD and Rietveld refinement patterns. **c** HAADF-STEM and **d** ABF-STEM image of Na_0.95_LNCFM at the [010] zone axis. **e, f** STEM-EDS Line sweep. **g** STEM-EDS Element mapping

Inspired by HEOs in electrochemical energy storage, we designed a novel five-component layered HEO O3-Na_0.95_Li_0.06_Ni_0.25_Cu_0.05_Fe_0.15_Mn_0.49_O_2_ as a cathode for SIBs, the schematic diagram is shown in Fig. 1a. The HEO cathode constructed by compatible radius and different Fermi level ions is effective for preventing charge ordering and reducing the electronic localization [12]. Meanwhile, ionic conductivity can be enhanced through disorder, which contributes to energetically favorable routes in high-entropy lattices [23]. Owing to the poor overlap between Li: 1s and O: 2p orbitals, the bonding between Li and O is predominantly ionic, resulting in an improved interaction of the TM center with oxygen orbitals and transfer of charge from sodium to oxygen. The strength of the *TM*-O and Na–O bonds for Na_0.95_Li_0.06_Ni_0.25_Cu_0.05_Fe_0.15_Mn_0.49_O_2_ enhances structure stability. Although ionic bonds are not as strong as covalent bonds, they repair themselves when broken. The combination of ionic and covalent bonds with hardness and softness alleviates stress. Moreover, the Li^+^ and Cu^2+^ with low valence doping increases the valence of manganese ions, which impedes the Jahn–Teller effect. The introduction of Na vacancies decreases the tetrahedral site energy via increased interlayer distance due to the decreased shielding effect [7, 24].

In consequence, this HEO O3-type cathode exhibits excellent rate performance and keeps highly reversible structure evolution during cycling, delivering a high reversible capacity of 111.4 mAh g^−1^ at 1600 mA g^−1^ and retaining capacity of 83.2% after 500 cycles. Even at 4000 mA g^−1^, this HEO O3-type cathode exhibits a capacity of 85.8 mAh g^−1^ and keeps retention of 85.1% after 1000 cycles, which is among the best of recently reported results for different O3 -type cathodes. The PSE battery assembled by this HEO cathode provides a capacity of 92.1 mAh g^−1^ at 5C and retains 96% of its capacity after 400 cycles. This work offers novel perspectives for the utilization of HEOs in Na-ion batteries.

## Experimental Section

### Materials

Sodium carbonate (Na_2_CO_3_, 99.5%), nickel oxide (NiO, 99%), manganic oxide (Mn_2_O_3_, 98%) and sodium bis(trifluoromethylsulfonyl)imide (NaTFSI) were purchased from Alfa Aesar (China) Chemical Co., Ltd. Iron (III) oxide (Fe_2_O_3_, 99.9%), cupric oxide (CuO, 99.9%), silica (SiO_2_, 20 nm) and poly(vinylidene fluoride-co-hexafluoropropylene) (PVDF-HFP) were purchased from Shanghai Macklin Biochemical Technology Co., Ltd. Lithium carbonate (Li_2_CO_3_, 99.998%) was purchased from Shanghai Aladdin Bio-Chem Technology Co., Ltd. Propylene carbonate and acetone were purchased from Sinopharm Group Co. Ltd. All chemicals were used without future purification.

### Materials Synthesis

#### Synthesis of Na_0.95_Li_0.06_Ni_0.25_Cu_0.05_Fe_0.15_Mn_0.49_O_2_

The O3-Na_0.95_Li_0.06_Ni_0.25_Cu_0.05_Fe_0.15_Mn_0.49_O_2_ materials were synthesized through a typical solid-state method from stoichiometric amounts of Na_2_CO_3_, Li_2_CO_3_, NiO, Fe_2_O_3_, CuO and Mn_2_O_3_. The mixtures were ground using a mortar and then ball-milled at 350 rmp for 5 h. After that, the obtained powders were pressed into pellets under pressure of 15 MPa and then calcined at 900 ℃ in muffle furnace for 24 h at a heating rate of 3 °C min^−1^. After cooling to room temperature, the obtained powders were transferred to a glove box filled with argon (H_2_O, O_2_ < 0.1 ppm) for storage.

#### Synthesis of PSE

To prepare PSE, PVDF-HFP (1.0 g), NaTFSI (0.47 g), SiO_2_ (0.1 g) and PC (2 mL) were dissolved in acetone (10 mL) under continuous stirring for 2 h at 60 °C. The obtained semitransparent sol was distributed on a glass plate, scraped to form a level membrane, and then dried at 60 ℃ for 20 min in an oven.

The detailed material characterizations and electrochemical measurements were provided in the Supporting Information.

## Results and Discussion

### Crystal Structure Characterization

HEO O3-Na_0.95_Li_0.06_Ni_0.25_Cu_0.05_Fe_0.15_Mn_0.49_O_2_ (Na_0.95_LNCFM) cathode was obtained by a typical solid-state reaction with a minor excess of 3% Na_2_CO_3_ ratio to minimize the presence of NiO impurities. The stoichiometry of Na_0.95_LNCFM was further confirmed by the inductively coupled plasma optical emission spectroscopic (ICP-OES). The obtained composition closely matches expectations (Table S1). The powder XRD patterns of sample indicate that the Na_0.95_LNCFM material exhibits a pure and highly crystalline O3-type α-NaFeO_2_ structure with R_m3 space group, confirming that entropy regulates solid solution formation by the decrease in Gibbs free energy. Precise crystallographic data are presented in Table S2. XRD patterns were excellently matched by using Rietveld refinement (Fig. 1b). Satisfied R-factors of *R*_p_ = 2.7% and *R*_wp_ = 3.5% were obtained.

The detailed atomic-scale structure of Na_0.95_LNCFM sample was further verified using aberration-corrected STEM. In Fig. 1c, the HAADF image exhibits a well-organized pattern of *TM* atoms along the [010] direction. The interlayer distance dc is measured to be ≈0.539 nm, which aligns with the powder XRD refinement results. The ABF image of Fig. 1d reveals that the oxygen layers are arranged in an ABCABC pattern along the [001] direction with Na–O octahedra positioned between *TM*O_2_ layers, indicating a layered O3 phase. As shown in Fig. 1e–f, the STEM–EDS line sweep shows that Ni, Cu, Fe and Mn elements together occupy the octahedral sites within transition metal layers. The STEM-EDS mapping (Fig. 1g) exhibits atomic-scale uniform distribution of Ni, Cu, Fe, Mn and O elements in the O3-Na_0.95_LNCFM. The SEM image of as-prepared cathode particles shows a spherical particle morphology with sizes of around ≈2 to 5 µm (Fig. S1). Moreover, SEM–EDS (Fig. S2) and TEM-EDS (Fig. S3) mappings demonstrate the uniform distribution of Na, Ni, Cu, Fe, Mn and O elements in the O3-Na_0.95_LNCFM entire particles, without a segregation phenomenon. Furthermore, X-ray photoelectron spectroscopy (XPS) was performed to explore the chemical states of various elements in the Na_0.95_LNCFM. Figure S4 shows the peaks of Cu 2*p*, Fe 2p, Li 1*s* and Na 1*s*. The peaks located at 50–70 eV are attributed to the Li-K-edge. The binding energy of Fe 2*p*, Cu 2*p* and Li 1*s* indicates that the valence states of corresponding elements are + 3, + 2, and + 1, respectively. The XPS spectrum of Ni 2*p* exhibits peaks associated with Ni 2*p*_3/2_ and Ni 2*p*_1/2_ at 854.7 and 872.2 eV, respectively, and corresponding satellite peaks [10, 25]. The presence of these peaks of Ni 2*p* demonstrate that oxidation states of Ni exist + 2 and + 3. The peaks in the Mn 2*p* XPS spectrum can be attributed the existence of Mn^3+^ and Mn^4+^ (Fig. S5) [10]. Due to the existence of Li^+^ and Cu^2+^ with low oxidation state in the *TM*O_2_ layers and the sodium vacancy, the average oxidation states of the Mn and Ni in Na_0.95_LNCFM are increased. The increase in Mn valence state alleviates the Jahn–Teller effect.

### Electrochemical Performance

**Fig. 2:**
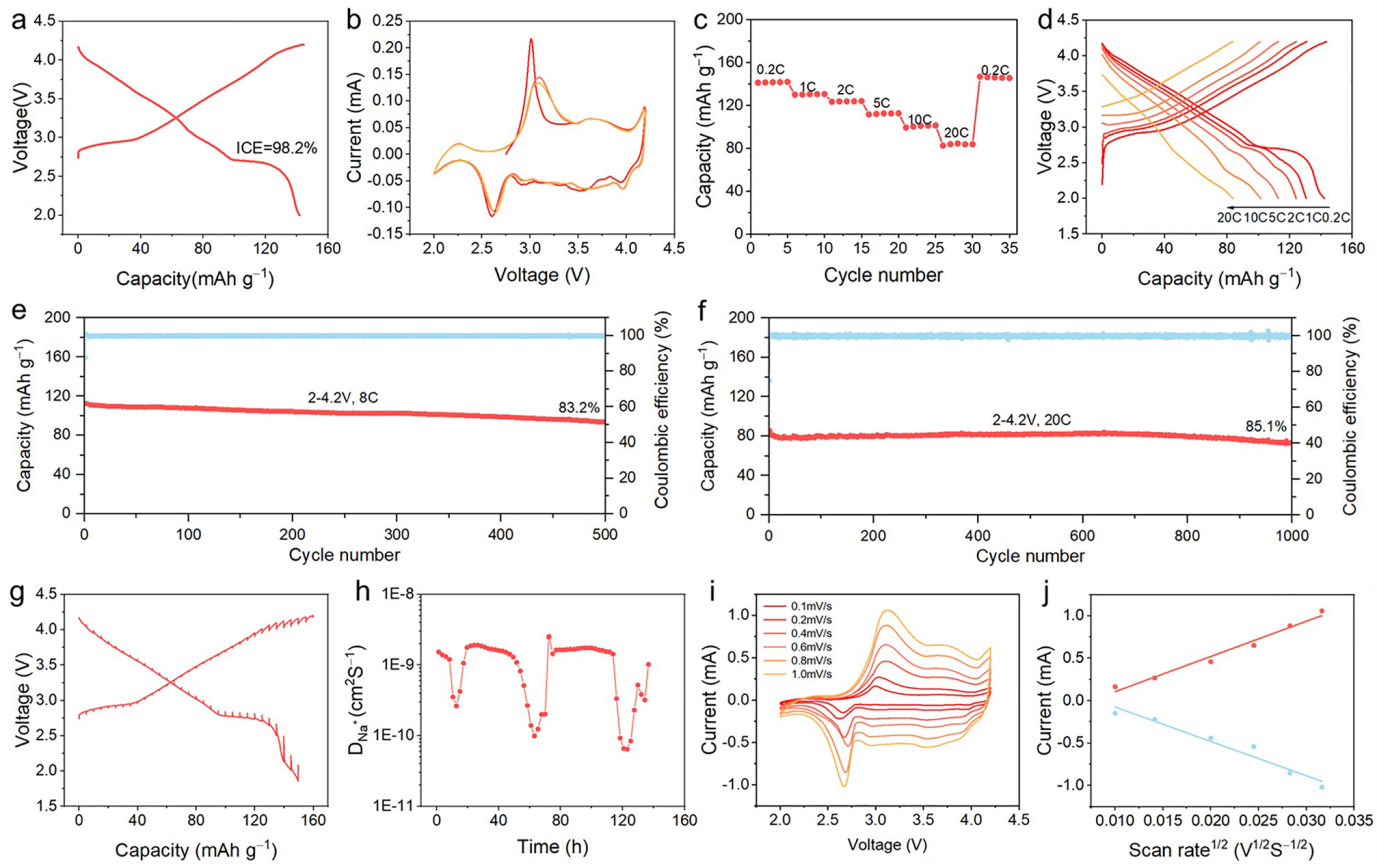
Electrochemical performance of Na_0.95_LNCFM cathode. **a** The initial charge − discharge curve at a 0.2C (1C = 200 mA g^−1^). **b** CV curves of the electrode scanned with 0.1 mV s^−1^. **c** Charge/discharge profiles at various rates. **d** Charge − discharge curves at various rates. **e, f** Long-term cycling performance at 8C rate and at 20C rate. **g** GITT curves. **h** The corresponding D_Na_^+^ calculated by GITT. **i** CV curves at different scan rates. **j** Liner relationship of peak currents with the scanning rate

The electrochemical characteristics of Na_0.95_LNCFM cathode materials were investigated in a sodium half-cell. Figure 2a illustrates the galvanostatic charge/discharge (GCD) curve of Na_0.95_LNCFM at a current density of 40 mA g^−1^ between 2.0 and 4.2 V. The cathode shows first reversible discharge capacity of 141.2 mAh g^−1^ with a high initial Coulombic efficiency (ICE) of ~ 98.2%. The charge/discharge profiles demonstrate a voltage plateau around 2.8 V and a sloping voltage profile within 2.8–4.2 V potential window. The sloping region is attributed to a solid solution reaction during the extraction and insertion of sodium. Meanwhile, the voltage plateau is likely a result of phase change between O3 and P3 phases [16, 26]. Figure 2b exhibits the CV curves of the Na_0.95_LNCFM electrode between 2.0 and 4.2 V versus Na^+^/Na at a scan rate of 0.1 mV s^−1^. The Na_0.95_LNCFM electrode exhibits consistent anodic/cathodic peaks at 3.05/2.60 and 3.63/3.58 V during repeated cycling, indicating the high reversibility. In Fig. 2c, d, the rate performance was measured and the reversible capacities are 141.7, 130.7, 124, 112.6, 101.3 and 83.5 mAh g^−1^ at 0.2C, 1C, 2C, 5C, 10C and 20C, respectively. After rate cycling, a reversible capacity of 146.8 mAh g^−1^ is achieved at a rate of 0.2C, demonstrating the exceptional reversibility of the Na_0.95_LNCFM cathode material. The cycling performance of the Na_0.95_LNCFM cathode is shown in Fig. S6. The Na_0.95_LNCFM electrode retains 88% of its capacity after 200 cycles at a current density of 400 mA g^−1^ with a high Coulombic efficiency. Moreover, the cathode exhibits long-term cycling stability under high rates. The Na_0.95_LNCFM cathode delivers a capacity of 111.4 mAh g^−1^ at 8C (1600 mA g^−1^) and keeps retention of 83.2% after 500 cycles (Fig. 2e). Even at 20C (4000 mA g^−1^), this cathode also provides a capacity of 85.8 mAh g^−1^ and keeps retention of 85.1% after 1000 cycles (Fig. 2f). The outstanding cycling and rate performance indicate that Na_0.95_LNCFM is a prospective cathode material for SIBs. Table S3 presents the electrochemical performance of previous published O3-type layered cathode materials, which enables us to make a contrast.

To obtain further comprehension of the excellent electrochemical property, galvanostatic intermittent titration technique (GITT) and CV measurement were conducted to explore the electrochemical kinetics behavior of Na_0.95_LNFM. According to the GITT profile of Na_0.95_LNCFM (Figs. 2g and S7–S8), the curve with low polarization is typical of the signature of solid solution behavior. The value of D_Na_^+^ is between 6.445 × 10^−11^ and 2.527 × 10^−9^ cm^2^ s^−1^ (Fig. 2h). The minimal value can be detected in the D_Na_^+^ against voltage plots which correlate to the charge/discharge plateaus, and generally represent the O3-type to P3-type phase transition. The D_Na_^+^ value of the second cycle varies from 5.126 × 10^−11^ to 2.46 × 10^−9^ cm^2^ s^−1^ (Fig. S9). To explore the change of electrochemical kinetics behavior along with the electrochemical cycle, we test the D_Na_^+^ after 20 cycles by GITT, which is shown in Fig. S10. The value of D_Na_^+^ is between 5.757 × 10^−11^ and 2.477 × 10^−9^ cm^2^ s^−1^, which is almost the same as the second cycle. Compared with Na_0.95_LNCFM, the D_Na_^+^ value of the Na-full NaLNCFM is between 2.415 × 10^−11^ and 2.453 × 10^−9^ cm^2^ s^−1^ (Fig. S11), which is less than that of the Na_0.95_LNCFM. Figure 2i displays the CV curves of the Na_0.95_LNCFM cathode at various scanning rates from 0.1 to 1.0 mV s^−1^. As shown in Fig. 2j, the peaks display a linear shift with the increase in scan rate, indicating a diffusion-controlled process of sodium-ion transport. The Na^+^ diffusion coefficient (D_Na_^+^) of this cathode is computed using the Randles–Sevcik equation. The computed values of D_Na_^+^ are 4.091 × 10^−11^ and 3.896 × 10^−11^ cm^2^ s^−1^ for the process of charge and discharge, respectively. Meanwhile, the diffusion coefficient exhibits excellent symmetry, indicating that sodium ion diffusions are highly reversible. More impressively, the density of states (DOS) of Na_0.95_LNCFM (Fig. S12) exhibits some metallic properties, confirming its desirable electronic conductivity. The excellent inherent electronic conductivity and diffusion coefficient provide the foundation of high-rate performance for Na_0.95_LNCFM.

### Structure Evolution and Reaction Mechanism

**Fig. 3:**
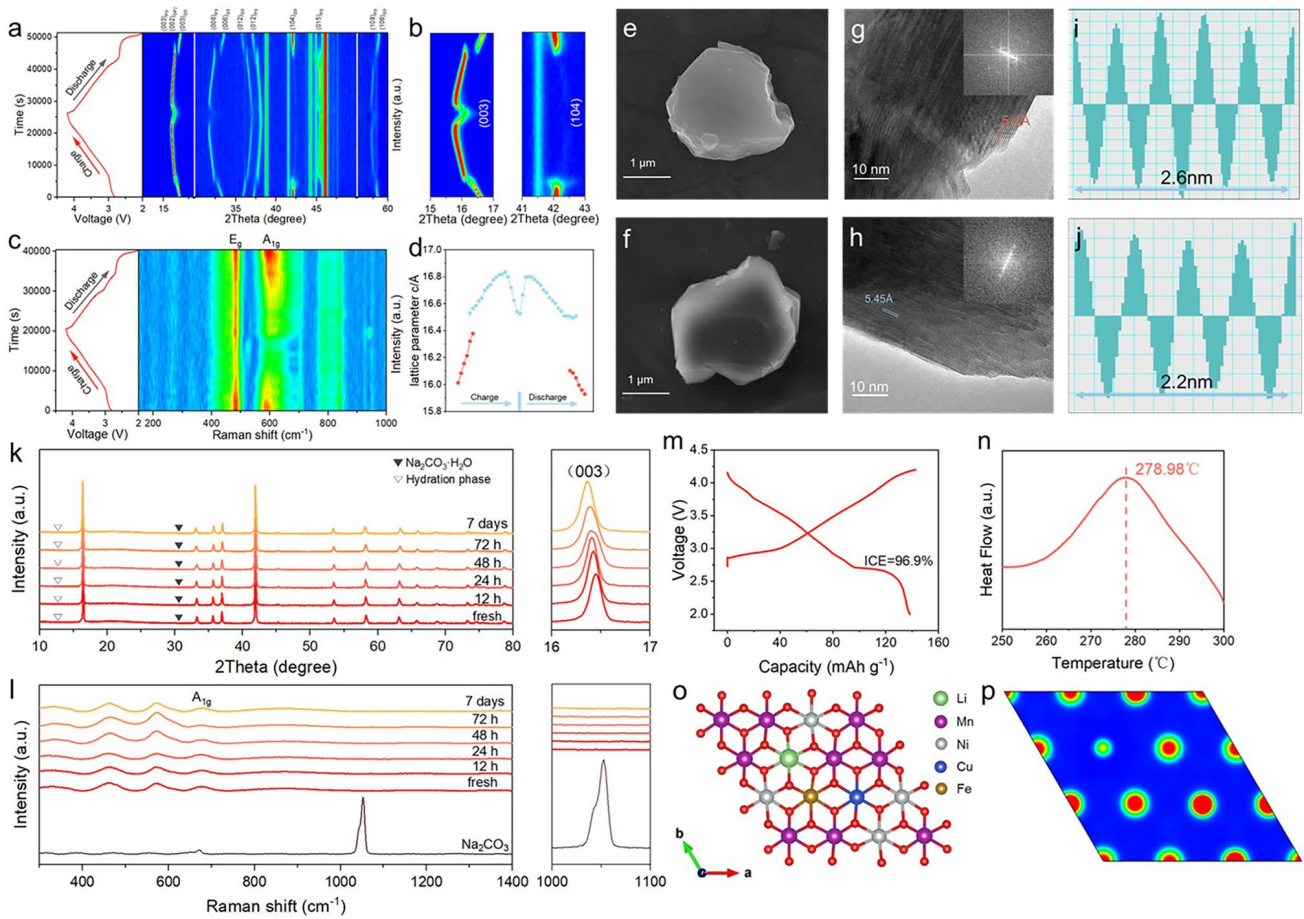
**a** In situ XRD patterns of Na_0.95_LNCFM during the first charge–discharge cycle at 20 mA g^−1^ between 2.0 and 4.2 V. **b** Contour map of in situ XRD patterns between 15°–17° and 41°–43°. **c** In suit Raman spectrum of Na_0.95_LNCFM during the first charge–discharge cycle at 25 mA g^−1^ between 2.0 and 4.2 V. **d** The lattice parameter c along with Na extraction/insertion for Na_0.95_LNCFM. **e** SEM image of Na_0.95_LNCFM before cycle.** f** SEM image of Na_0.95_LNCFM after 50 cycles. **g** The HRTEM image of Na_0.95_LNCFM. **h** The HRTEM image of Na_0.95_LNCFM after 50 cycles. **i** Intensity profile corresponding to **g**. **j** Intensity profile corresponding to** h**. **k** The XRD patterns of initial and exposed Na_0.95_LNCFM and corresponding magnified region of 16°–17° on the right. **l** The Raman spectrum of pristine and exposed Na_0.95_LNCFM corresponding magnified region of 1000–1100 cm^−1^ on the right. **m** The initial charge − discharge curve at a 0.2C after 48 h exposure. **n** DSC profiles of Na_0.95_LNCFM cathode charged to 4.2 V. **o** Schematic structures of Na_0.95_LNCFM.** p** Contour maps of charge density on corresponding planes in Na_0.95_LNCFM

To reveal the fundamental mechanism responsible for exceptional cycling stability and high-rate performance of Na_0.95_LNCFM, in situ XRD measurement was performed during the first charge/discharge cycle at 0.1C. As shown in Fig. 3a-b, during first extraction of Na^+^, the (003) and (006) peaks experience a shift toward lower angle. Conversely, the (104) peak undergoes a movement toward higher angle. Upon further desodiation, the (003) and (006) peaks separate into two, while the strength of (104) peak noticeably decreases. This suggests the disappearance of original O3-type phase and the emergence of a new P3-type phase [7, 27]. As the (104) peak completely vanishes, a solid-solution reaction of P3 phase occurs until charged to 4.05 V without the appearance of any new peaks. As the removal of Na^+^, a distinct OP2 phase emerges, accompanied by a reversible phase transition P3 to OP2. This transition is observed within the 4.05–4.2 V potential window. During the discharge process, the crystal structure exhibits an opposite OP2–P3–O3 evolution, demonstrating that the structure transition is highly reversible at high voltage. The high-entropy design contributes to the excellent structural reversibility, in particular, the introduction of Li^+^ ions mitigates the in-plane electrostatic repulsion among cations and breaks the cation ordering. The transition metal cation ordering leads to Na^+^/vacancy ordering which is typically harmful for long-term stability and rate performance. Significantly, the P3 phase with wider interlayer spacing and open Na migration channel is maintained throughout a broad capacity range of 86.8%, offering a powerful support for the high-rate performance of Na_0.95_LNCFM. Figure 3d illustrates the change of crystal parameter c at various charge/discharge stages extracted from the in situ XRD patterns. The change of c-spacing value during sodiation and desodiation process is only 5.1% and the deviation of c-spacing during the initial cycle is only 0.52%. The minor volume change of Na_0.95_LNCFM is ascribed to the transition to P3 phase at low voltage regions and the initial sodium vacancy in the lattice. Furthermore, in situ Raman spectra (Fig. 3c) also directly verifies the reversible sodiation and desodiation process of Na_0.95_LNCFM. The peaks located at 569 and 459 cm^−1^ are identified as the A_1g_ and E_2g_ vibrational mode, respectively [28]. In addition, the A_1g_ and E_2g_ vibrational modes are credited to the transition metal–oxygen (*TM*-O) and sodium-oxygen (Na–O). The A1g vibrational mode (Na–O) significantly weakens during charging, then becomes enhanced upon discharging. The E_2g_ vibrational mode remains even at 4.2 V, which can be attributed to the Li and Cu dopants. The Li dopant increases the valence state of Mn, which further improves the bonding energy between Mn and O, hence contributing to stabilization of the structure throughout the process of Na-(de)intercalation. Meanwhile, the Cu dopant with high coordination ability acts as a rivet to impede the irreversible fracture of TM-O and O-TM-O bonds and further prevents a severe structural damage at high voltage [29].

To verify the structural integrity of Na_0.95_LNCFM after long-term cycling, a series of characterizations by SEM, XRD, Raman and TEM were performed to provide the information about the structure evolution [30]. As displayed in Figs. 3e-f and S13, the SEM images of the cycled Na_0.95_LNCFM cathode show that the particle is intact without crack even damage, and only has slight expansion of layers. As displayed in Fig. S14, the XRD pattern reveals the reversibility of structure, the structure remains O3 phase after 50 cycles and has no transition metal dissolution (Fig. S15). The HRTEM image and diffraction information along the [001] zone axis of cycled material (Fig. 3g–j) exhibit that the structure remains O3 phase and the interslab distance increases from 5.4 to 5.45 Å. Therefore, such strategy suppresses the phase transition and volume change during the sodiation and desodiation process.

### Thermal Stability and Air Stability

In order to achieve the practical applications of NIBs, the thermal stability and air stability are two crucial indicators, which are related to the safety requirements and storage costs. To reveal the structure–composition relationship between the initial and air exposed Na_0.95_LNCFM materials, a series of characterizations by XRD, Raman, SEM and charging capacities was conducted to provide the information. As displayed in Fig. 3k, the Na_0.95_LNCFM remains an O3 α-NaFeO_2_ structure and does not have formation of hydration phase and Na_2_CO_3_·nH_2_O after seven days exposure. The (003) peak of Na_0.95_LNCFM slightly shifts to a lower angel, demonstrating that the Na content in the lattice has a slight reduction instead of phase transition. Furthermore, optical Raman spectroscopy measurement was carried out to study the structural change in the Na_0.95_LNCFM. Meanwhile, optical Raman spectroscopy is sensitive to C–O and C=O bond, therefore, it can detect the formation of Na_2_CO_3_ under exposure. As shown in Fig. 3l, three characteristic peaks are observed from the freshly prepared Na_0.95_LNCFM at ∼460, 573 and 679 cm^−1^, which are ascribed to Ni–O, Mn–O and Na–O stretching. The characteristic peak at 1052 cm^−1^ is attributed to Na_2_CO_3_. The Ni–O and Mn–O peaks remain strong along the increase in exposure time, which indicates excellent structure stability. The Na–O peak almost remains the same intensity during the air exposure process, indicating that Na^+^ ions retain in the lattice. In addition, the peak at 1052 cm^−1^ does not obviously appear, indicating that the particle surface of Na_0.95_LNCFM has no significant deposition of Na_2_CO_3_. Furthermore, the particle morphology of Na_0.95_LNCFM after air exposure was identified by SEM (Fig. S16). The morphology of particle remains intact without serious crack and fiber-like particles. Meanwhile, the surface of particle has not the formation of residual alkali impurity. In general, the structure transformation under air exposure leads to the notable reduction of discharge capacity. Therefore, we used the cathode materials after 48 h exposure to assemble coin cell to examine electrochemical performance. As shown in Fig. 3m, the discharge capacity of the first cycle is 138.2 mAh g^−1^ with an initial Coulombic efficiency (ICE) of ~ 96.9%. Meanwhile, the air exposure electrode exhibits a high capacity retention of 83.7% after 200 cycles at a current density of 400 mA g^−1^ (Fig. S17), which is slightly less than the initial cathode material. Differential scanning calorimetry (DSC) as an efficient method was employed to identify the thermal impact on cathode thermal stability [19]. The electrode was assessed at the charged state of 4.2 V and with the presence of an electrolyte (electrolyte-to-cathode mass ratio of 2:1). As shown in Figs. 3n and S18, the obvious exothermic peak is mainly caused by the liberation of oxygen from the crystal structure and reduction of active *TM* upon heating, which results in the phase transition from the OP2 phase to the *TM*O_2_ structure followed by the creation of heat [26, 31]. The Na_0.95_LNCFM cathode exhibits an exothermal peak at around 279 ℃, which demonstrates that the Na_0.95_LNCFM has good thermal safety. In order to further get a deeper comprehension of the reason for excellent air stability and thermal stability of Na_0.95_LNCFM electrode, DFT calculations were conducted to explore the intrinsic electronic structure. The calculation model of Na_0.95_LNCFM is shown in Fig. S19. As displayed in Fig. 3o–p, the charge density of Li^+^ is weaker than *TM* ions and the charge density of Cu^2+^ is stronger than Ni and Mn ions. The Li^+^ ions substitution with slightly larger radius and different Fermi level constructs a weaker hybridization of Li–O orbital, which promotes the transfer of charge from sodium to oxygen to form a stronger binding energy of Na–O [32]. The stronger Na–O binding energy is beneficial to alleviate the spontaneous active Na loss and restrain the irreversible phase transition under air exposure. Meanwhile, the Cu^2+^ with a high electrochemical redox potential is more difficult to oxidize under air exposure and extract Na^+^ from the lattice chemically [33].

### Charge Compensation Mechanism

**Fig. 4:**
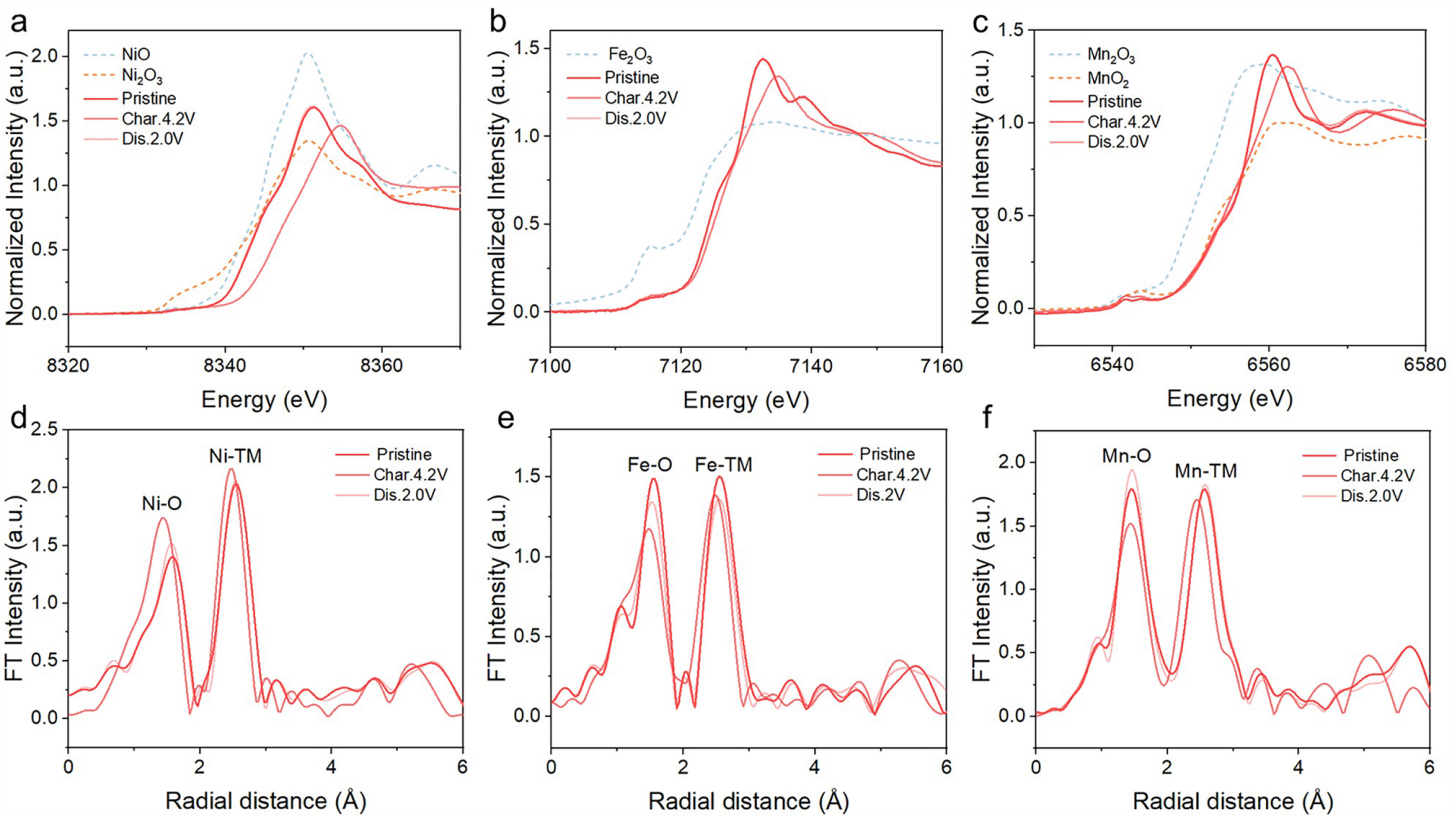
Ex situ XANES spectra at **a** Ni, **b** Fe and **c** Mn K-edge of Na_0.95_LNCFM at different charge/discharge states. Corresponding ex situ EXAFS spectra at **d** Ni, **e** Fe, and **f** Mn K-edge of N_0.95_LNCFM electrodes at different charge/discharge states

In order to gain insight into the charge compensation mechanism and the structure change during the charge/discharge process, Ex situ X-ray absorption spectroscopy (XAS) spectra were obtained at K-edge of Ni, Fe and Mn. Figure 4a–c exhibits the normalized X-ray absorption near edge structure (XANES) spectra of Ni, Fe, and Mn K-edges. Additionally, the spectra of the respective metal oxide powders are included as standard references. When charged to 4.2 V, the Ni K-edge XANES spectra exhibit a notable shift toward higher energy. This shift is indicative of the redox process from Ni^3+^ to Ni^4+^. When discharged to 2.0 V, the Ni K-edge XANES spectra return to the original location, indicating that the redox process of Ni is electrochemically reversible. Similarly, the Fe K-edge XANES spectra exhibit obvious shift toward higher energy as charge increases to 4.2 V. This shift demonstrates that Fe undergoes oxidation to a higher valence. Upon further discharge, the Fe K-edge XANES spectra return to the original position. However, slight shift of Mn K-edge is observed, which can be attributed to the local environment changes surrounding Mn during the Na^+^ (de)intercalation process. Hence, the charge compensation originates from the oxidization/reduction of Ni and Fe ions. Meanwhile, few Mn^4+^/Mn^3+^ redox is advantageous for mitigating the Jahn–Teller effect. Figure 4d–f displays the corresponding extended X-ray absorption fine structure (EXAFS) spectra. The two prominent peaks in the Ni, Fe, and Mn K-edges are correspond to the average TM-O length of the first shell TM-O_6_ configuration and the TM–TM length of the second shell TM–TM_6_, respectively [18, 34, 35]. As displayed in Fig. 4d-e, Ni–O, Fe–O and Mn–O bonds length undergo a reversible decrease/increase throughout the charge/discharge process. All results indicate that the Na_0.95_LNCFM possesses excellent structural stability, aligning with the information obtained from in situ XRD.

### Configuration Toward Practical Applications

**Fig. 5:**
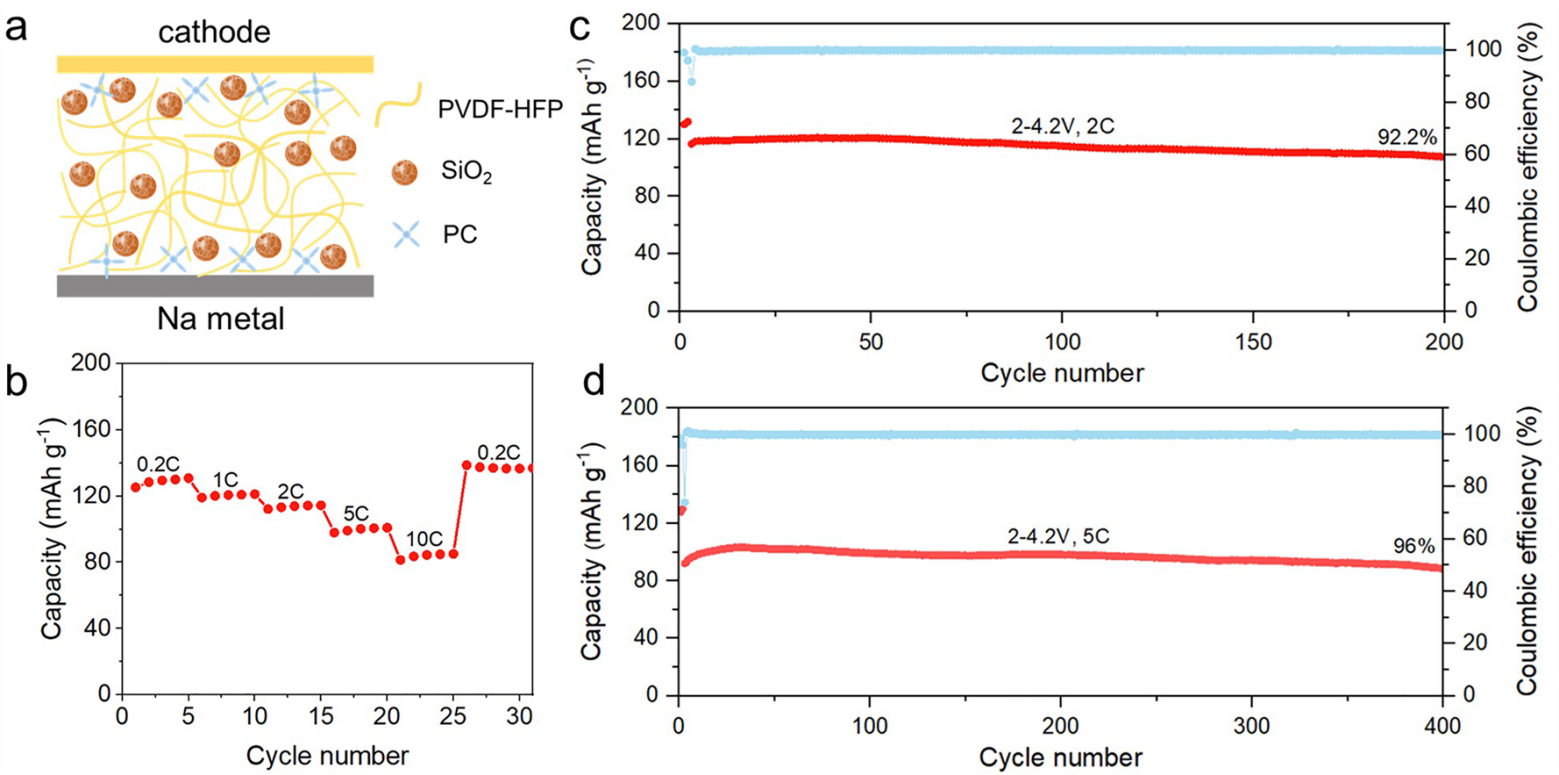
Electrochemical performance of Na_0.95_LNCFM PSE battery. **a** Schematic illustration for Na_0.95_LNCFM PSE battery. **b** Rate performance. Charge/discharge profiles at various rates. **c, d** Long-term cycling performance at 2C rate and at 5C rate

To verify the application of the cathode material in solid-state SIBs, polymer solid-state sodium battery is assembled. As displayed in Fig. 5a, the electrochemical performance of Na_0.95_LNCFM was measured in the button cell using polyvinylidene fluoride hexafluoropropylene (PVDF-HFP)-based gel as polymer solid electrolyte (PSE). The PVDF-HFP-based PSE consists of PVDF-HFP, propylene carbonate (PC) and aerosol (nano-silica). The PVDF-HFP-based QSE with the dispersion of small holes and a thickness of ≈100 μm (Fig. S21) might be beneficial for the infiltration of electrolyte and the Na^+^ conduction [36]. The electrochemical stability voltage reaches over 4.8 V and ionic conductivity is calculated to be 5.85 × 10^−4^ S cm^−2^ (Fig. S22). Furthermore, the QSE possesses good mechanical stability and toughness (Fig. S20). The galvanostatic charge/discharge (GCD) curves of Na_0.95_LNCFM PSE battery at a current density of 40 mA g^−1^ between 2.0 and 4.2 V are displayed in Fig. S23. The battery shows first reversible discharge capacity of 126.6 mAh g^−1^ with a high initial Coulombic efficiency (ICE) of ~ 98.8%. As displayed in Fig. 5b, the rate performance was measured and the reversible capacities are 130.9, 121.2, 114.5, 100.9, and 85.1 mAh g^−1^ at 0.2C, 1C, 2C, 5C and 10C, respectively. The cycling performance of the Na_0.95_LNCFM PSE battery is shown in Fig. 5c. The Na_0.95_LNCFM PSE battery delivers a capacity of 116.5 mAh g^−1^ at 2C (400 mA g^−1^) and keeps retention of 92.2% after 200 cycles. Even at 5C (1000 mA g^−1^), this battery also exhibits a capacity of 92.1 mAh g^−1^ and retains 96% of its capacity after 400 cycles (Fig. 5d). The outstanding cycling and rate performance of Na_0.95_LNCFM PSE battery is almost comparable to those of the Liquid battery, demonstrating a promising practical application of SIBs.

## Conclusion

In summary, we report a novel high-entropy O3-type layered cathode for solid-state sodium-ion batteries. The design of high-entropy structure and Li–TM interaction alleviate lattice stress and enhance ionic conductivity, enabling a rapid and reversible O3–P3 phase transition at low voltage regions and suppressing phase transition, thus leading to excellent rate and cycling performances. Meanwhile, due to the strengthened TMO_2_ framework and Na–O binding energy, Na_0.95_LNCFM exhibits remarkable air stability and thermal stability. The combination of high-entropy and Li–TM interaction conceptions is an effective strategy to regulate the phase evolution, air stability and thermal stability. Therefore, we believe that such strategy may also be extensively applied to a series of cathodes to improve the performance of solid-state Na-ion batteries.

## Supplementary Information

Below is the link to the electronic supplementary material.

Supplementary file1 (PDF 1998 kb)
