# Supplementary material for: High-Entropy Layered Oxide Cathode Enabling High-Rate for Solid-State Sodium-Ion Batteries
Source: Nanomicro Lett. 2023 Nov 9;16:10. doi: 10.1007/s40820-023-01232-0 (PMC10635981; doi:10.1007/s40820-023-01232-0)
Supplement: Supplementary file 1 — Supplementary file1 (PDF 1998 kb) [file 40820_2023_1232_MOESM1_ESM.pdf]

Supporting Information for

High-Entropy Layered Oxide Cathode Enabling High-Rate for Solid-State Sodium-Ion Batteries

Tianxun Cai[1,3], Mingzhi Cai[2], Jinxiao Mu[1,3], Siwei Zhao[2], Hui Bi[1], Wei Zhao[1,4], Wujie Dong[1], and Fuqiang Huang[1,2,3,*]

[1] State Key Laboratory of High Performance Ceramics and Superfine Microstructure, Shanghai Institute of Ceramics, Chinese Academy of Sciences, Shanghai 200050, P. R. China

[2] State Key Laboratory of Rare Earth Materials Chemistry and Applications, College of Chemistry and Molecular Engineering, Peking University, Beijing 100871, P. R. China

[3] Center of Materials Science and Optoelectronics Engineering, University of Chinese Academy of Sciences, Beijing 100049, P. R. China

[4] Zhongke Institute of strategic emerging materials, Yixing, Jiangsu 214213, P. R. China

* Corresponding author. E-mail: huangfq@mail.sic.ac.cn (Fuqiang Huang)

S1 Experimental Methods

S1.1 Materials Characterization

The crystal structure and morphology of the as-prepared samples were acquired by X-ray diffractometer on a Bruker D8 Advance diffractometer with Cu Kα X-ray beam source in the reflection mode in the 2θ range between 10° and 80°. The Fullprof software was used to analyze the crystal structure based on the Rietveld refinement method. The in-situ XRD test is performed in a specially designed Swagelok unit with the Be window covered by Al foil as the X-ray window and were periodically recorded every 20 min from 10° to 60° between 2.0 and 4.2 V at a current density of 20 mA g^{-1} during the first charge and discharge. The Raman spectroscopy was conducted on a Horiba LabRAM HR Evolution spectrometer with a laser excitation wavelength of 532 nm. The X-ray photoelectron spectra (XPS) of the original samples was gathered on ESCALab 250Xi (Thermo Scientific) spectrometer instrumented using an Al Kα X-ray beam. The binding energies of all elements were carefully calibrated on the basis of C 1s spectra (284.8 eV) and then Avantage software was used to accurately fit the XPS peak. The different elements concentration of $Na_{0.95}$LNCFM electrode was conducted on an inductively coupled plasma optical emission spectrometry (ICP-OES, Agilent 5110). Under 10 KV operating voltage, the scanning electron microscope (SEM) (Verios G4) was used to analysis the dimensions and morphology materials. The

interlayer structure and element distribution of the materials were identified by high-resolution transmission electron microscopy (HRTEM) and energy-dispersive X-ray spectroscopy (EDS) by taking advantage of TEM (JEM-F200) equipped with an EDS detector at an operating voltage of 200 KV. STEM-HAADF, ABF, EDS mapping were conducted on an aberration-corrected FEI-Titan Cubed Themis G2 300 scanning transmission electron microscope operating at 300 kV. The sample inspected by STEM was thinned to less than 100 nm using a 2–30 kV Ga ion beam on a dual-bean Helios Nanolab 460HP FIB-SEM system. For the thermal stability experiments, electrodes were first charged to 4.2 V versus Na metal and disassembled to collect the cathodes in an Ar-filled glovebox. And then the electrodes were washed with dimethyl carbonate before vacuum drying. An aluminum sealed pan was used to accommodate 2-3 mg dry electrodes with fresh electrolyte (1 M $NaClO_4$ in PC with 5% FEC, electrolyte-to-cathode mass ratio of 2:1). Differential scanning calorimetry (DSC) measurements were conducted by STA449F3 Jupiter at a temperature scan rate of 10°C min^{-1}. XAS measurements were carried out at beamline BL11B of the Shanghai Synchrotron Radiation Facility (SSRF), China, operating with a Si (111) double-crystal monochromator.

S1.2 Electrochemical Measurement

A slurry consisting of active material, super P as well as polyvinylidene difluoride (PVDF) with a weight ratio of 80:10:10 were dissolved in N-methylpyrrolidinone (NMP), then coated on aluminum foil to prepare working electrode. Then, the materials were separated from the solution and dried in an oven at 100°C for 10h and transferred to an Ar filled glovebox. The loading mass of the active material is 2-3 mg cm^{-2}. The electrochemical performances were tested by assembling a coin-type (CR2016) cell with pure sodium foil as the counter electrode in an argon-filled glove box. The electrolyte was 1M $NaClO_4$ dissolved in PC solvent with 5% FEC as an additive, and separator using by porous glass fiber (GF/D). The amount of electrolyte was controlled at around 200 µL. Galvanostatic discharge/charge was accomplished on a battery cycler (LAND CT-2001A, China) within potential windows of 2.0-4.2 V. Cyclic voltammetry (CV) measurements were conducted at a scan rate of 0.1 mV s–1 on a CHI760e electrochemistry workstation within potential windows of 2.0-4.2 V.

S1.3 Sodium-ion Diffusion Kinetics Calculation

For the galvanostatic intermittent titration technique (GITT) measurements, the cells were charged repeatedly at a current density of 20 mA g^{-1} over a period of 15 minutes to reach 4.2V, followed by open-circuit relaxation for 120 min. The detailed calculation formula is as follows:

$$D_{Na^+} = \frac{4}{\pi\tau}\left(\frac{m_B V_m}{M_B S}\right)^2 \left(\frac{\Delta E_s}{\Delta E_t}\right)^2$$

When the working current is very small, the above formula is applicable. Where the relaxation time is represented by τ; MB and mB are the molecular weight and mass

of the host oxide; The molar volume of the electrode material is represented by Vm; The contact area of the electrode/electrolyte is represented by S; The voltage change caused by the pulse is represented by ΔEs; The constant current charging/discharging voltage Variety is represented by ΔEt.

Moreover, cyclic voltammetry (CV) measurements of electrode between 2.0 and 4.2 V at different scan rates (0.1, 0.2, 0.4, 0.6, 0.8 and 1.0 mV s^{-1}) were be conducted, which demonstrates the redox characteristics is relevant to phase transition. The Na+ apparent diffusion coefficient was calculated according to the Randles-Sevcik Equation as followed:

$$i_p = (2.69 \times 10^5) n^{3/2} A D_{Na^+}{}^{1/2} C_{Na^+} v^{1/2}$$

Where n represents the number of electrons per reaction species, A represents the effective contact between electrolyte and electrode, D_{Na^+} is the diffusion coefficient of Na^+, and C_{Na^+} is the concentration of Na^+ in the lattice.

S1.4 DFT Calculations

The effect of high-entropy on the electronic and crystal structure was simulated by DFT calculations with the Vienna ab initio simulation package (VASP). The projector augmented wave (PAW) method and generalized gradient approximation (GGA) with Perdew–Burke–Ernzerhof (PBE) functional and GGA+U extension were used. The U values applied for Ni, Fe, Mn, Cu were 6.1, 4.5, 4.0 and 4.0 eV, respectively. An energy cutoff of 520 eV was used for the plane wave expansion of valence electron wave functions. The convergence criterion of the total energy was 10^{-5} eV/atom and the residual Hellmann-Feynman force was 0.02 eV/Å. The Gamma k-point sampling was used, and k-mesh was denser than 0.03 $Å^{-1}$. The simulations for bulk structure were performed on a 3 × 3 × 1 supercell including 27 formula units with 2 Li ions, 7 Ni ions, 4 Fe ions, 1 Cu ion and 13 Mn ions ($Na_{25}Li_2Ni_7Fe_4Cu_1Mn_{13}O_{54}$, 106 atoms in total).

Supplementary Figures

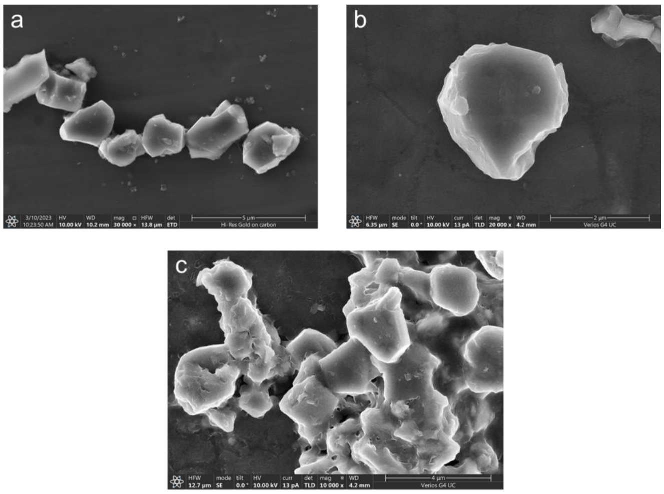

Fig. S1 SEM images of $Na_{0.95}Li_{0.06}Ni_{0.25}Cu_{0.05}Fe_{0.15}Mn_{0.49}O_2$

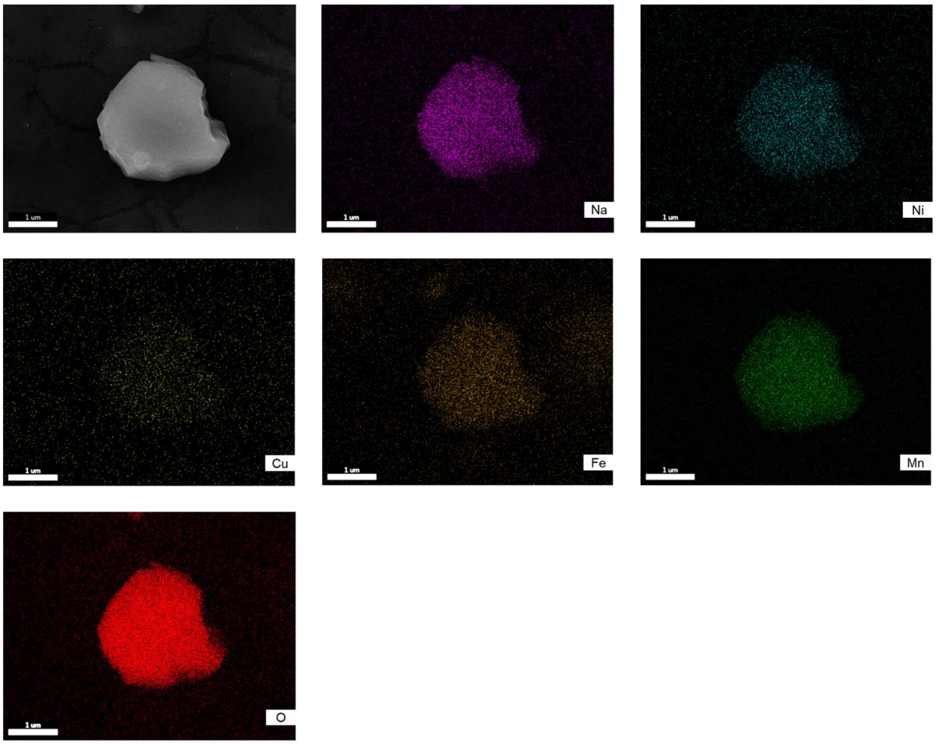

Fig. S2 SEM-EDS mapping of $Na_{0.95}Li_{0.06}Ni_{0.25}Cu_{0.05}Fe_{0.15}Mn_{0.49}O_2$

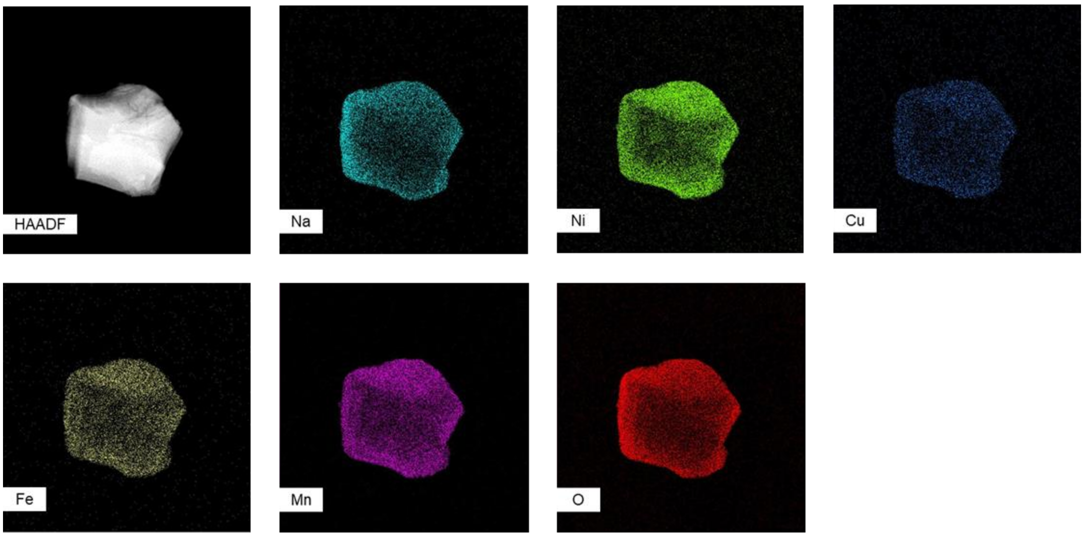

Fig. S3 TEM-EDS mapping of $Na_{0.95}Li_{0.06}Ni_{0.25}Cu_{0.05}Fe_{0.15}Mn_{0.49}O_2$

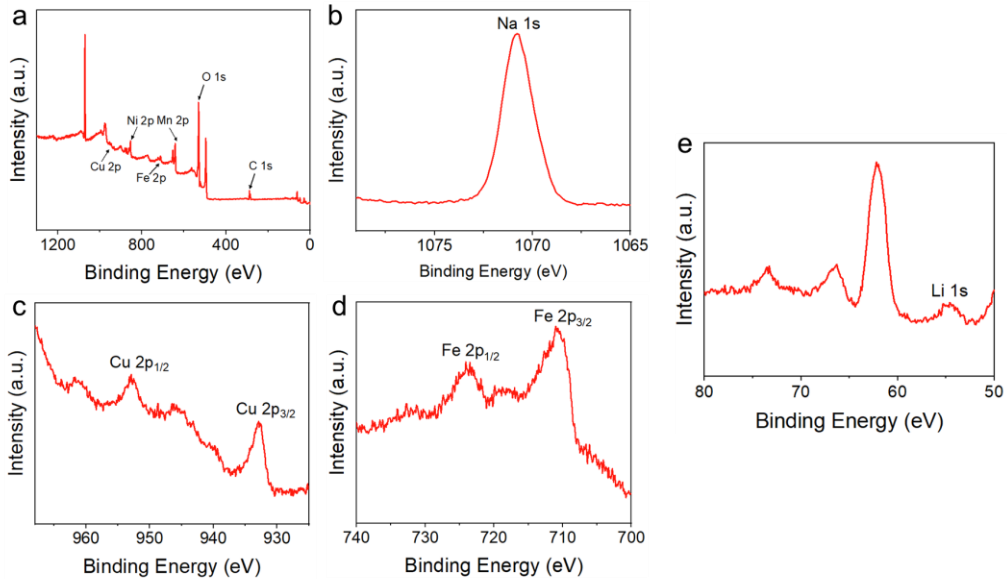

Fig. S4 The XPS spectra of $Na_{0.95}Li_{0.06}Ni_{0.25}Cu_{0.05}Fe_{0.15}Mn_{0.49}O_2$ for **a** XPS full spectrum analysis, **b** Na 1s region, **c** Cu 2p region, **d** Fe region and **e** Li 1s region

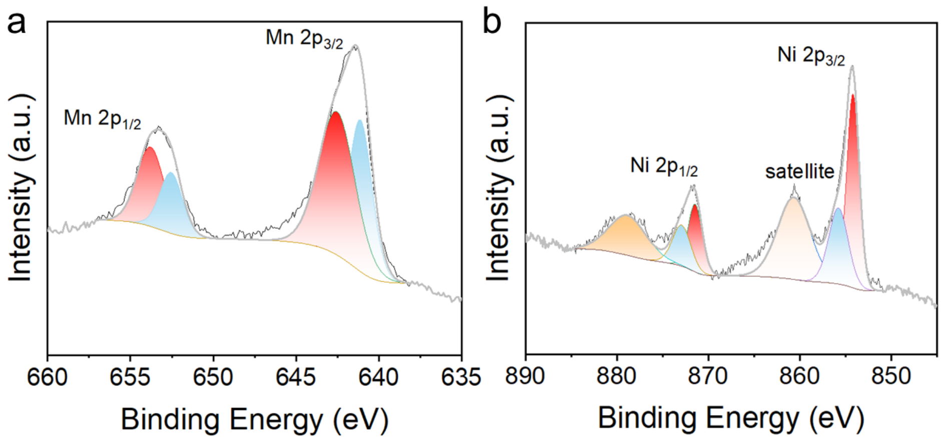

Fig. S5 The XPS spectra of $Na_{0.95}Li_{0.06}Ni_{0.25}Cu_{0.05}Fe_{0.15}Mn_{0.49}O_2$ for **a** Mn 2p region and **b** Ni 2p region

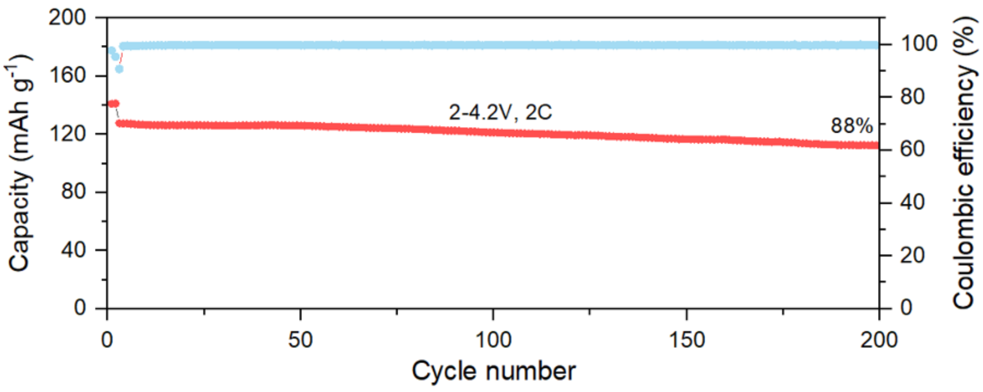

Fig. S6 Long-term cycling performance of $Na_{0.95}Li_{0.06}Ni_{0.25}Cu_{0.05}Fe_{0.15}Mn_{0.49}O_2$ at 2C (1C=200 mA g^{-1})

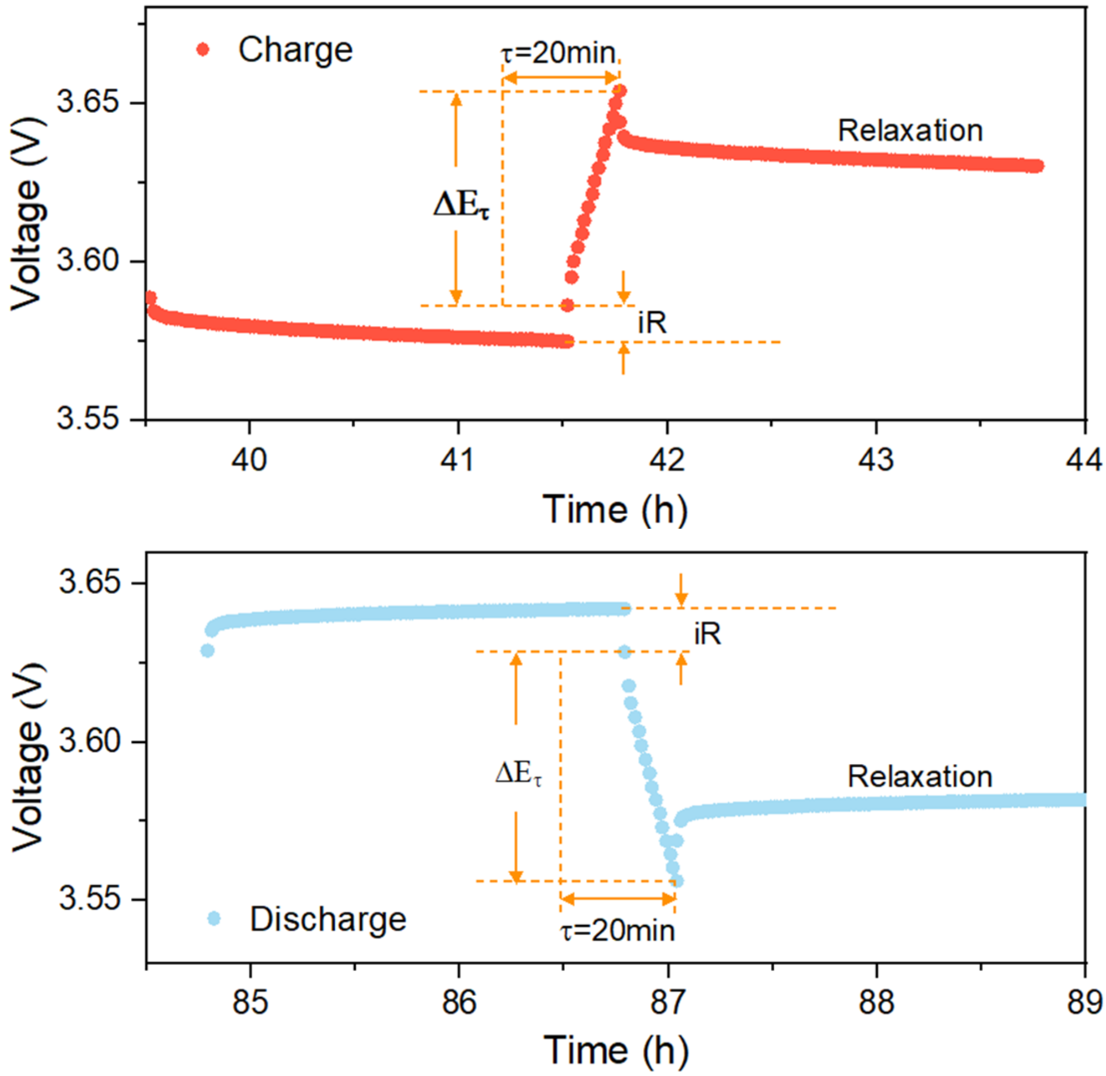

Fig. S7 One typical charge/discharge cycle of voltage profiles during the GITT measurement of $Na_{0.95}$LNCFM cathode

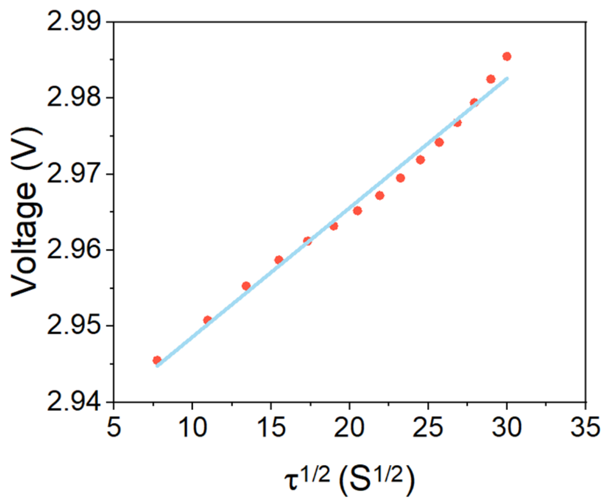

Fig. S8 Plot of voltage vs. $\tau^{1/2}$ to show the linear fit

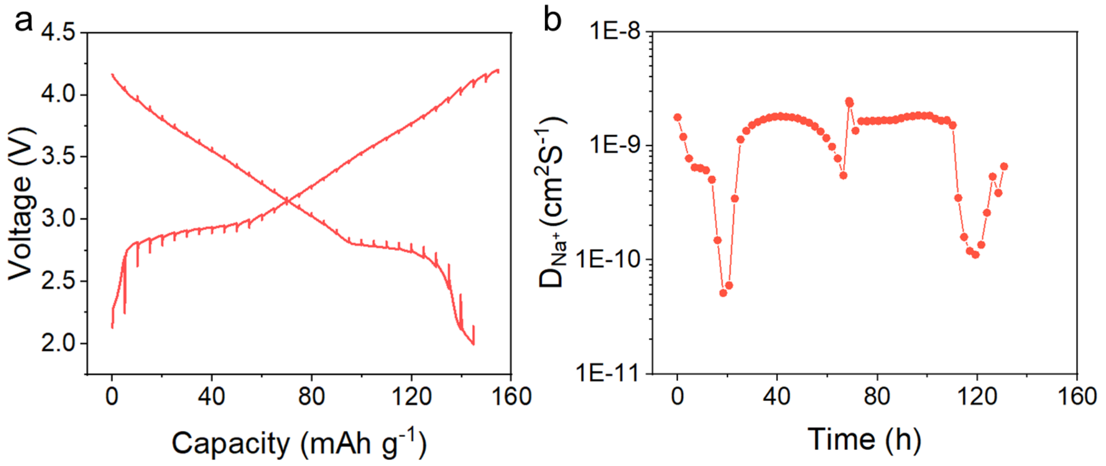

Fig. S9 a GITT curves for the charge and discharge states of the second cycle. **b** The corresponding $D_{Na}{}^{+}$ calculated by GITT

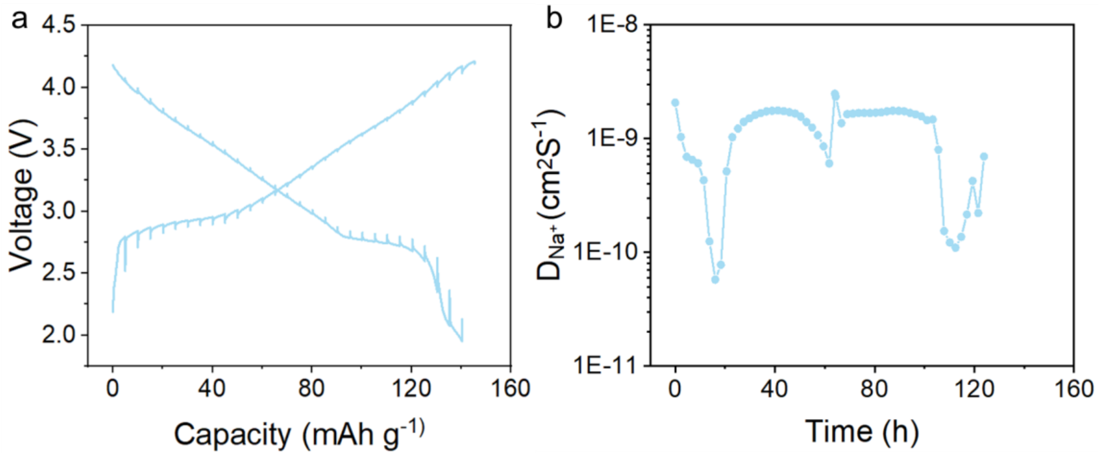

Fig. S10 a GITT curves for the charge and discharge states of the 21st cycle. **b** The corresponding $D_{Na}{}^{+}$ calculated by GITT

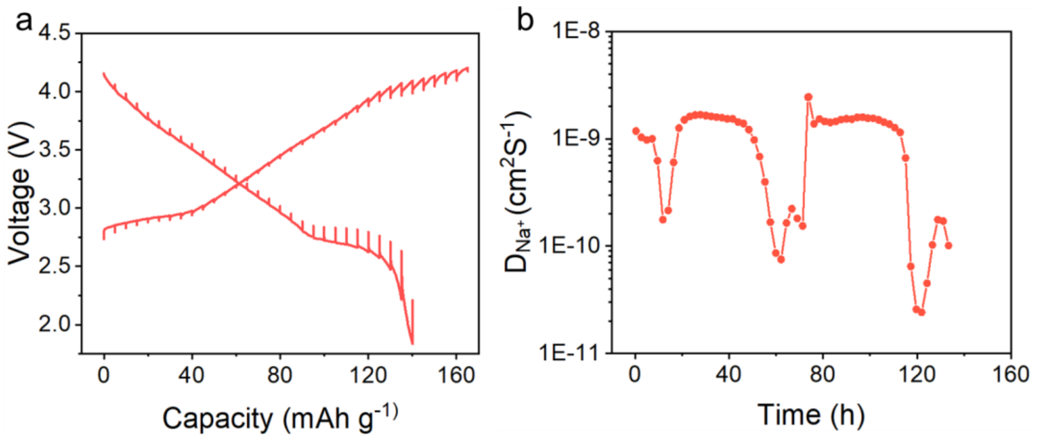

Fig. S11 a GITT curves of NaLNCFM during first cycle. **b** The corresponding $D_{Na}{}^{+}$ calculated by GITT

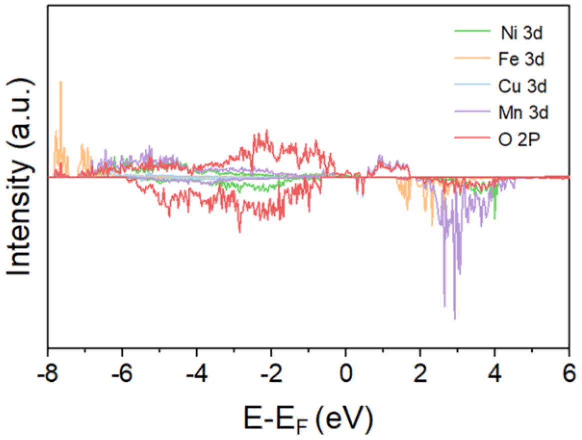

Fig. S12 Density of states of the $Na_{0.95}Li_{0.06}Ni_{0.25}Cu_{0.05}Fe_{0.15}Mn_{0.49}O_2$

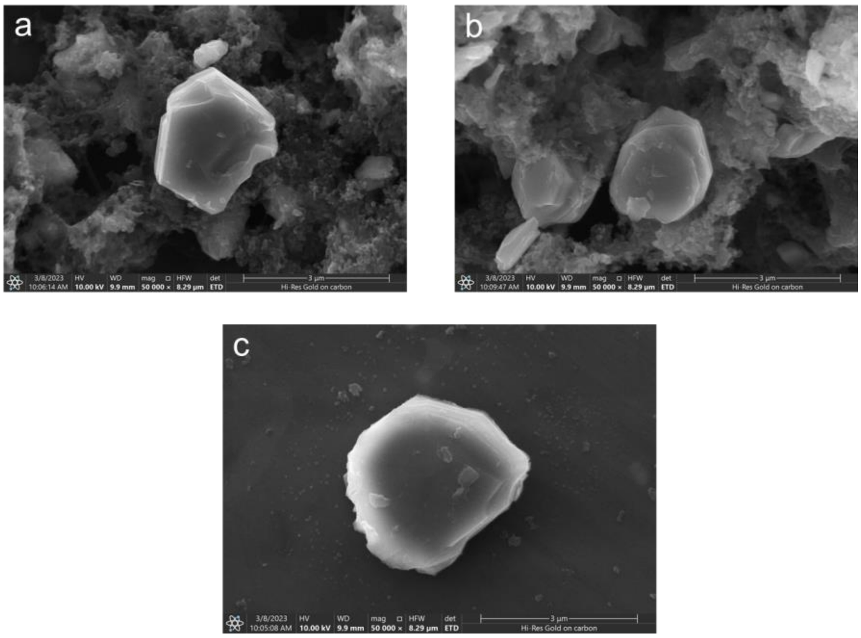

Fig. S13 SEM images of $Na_{0.95}Li_{0.06}Ni_{0.25}Cu_{0.05}Fe_{0.15}Mn_{0.49}O_2$ after 50 cycles

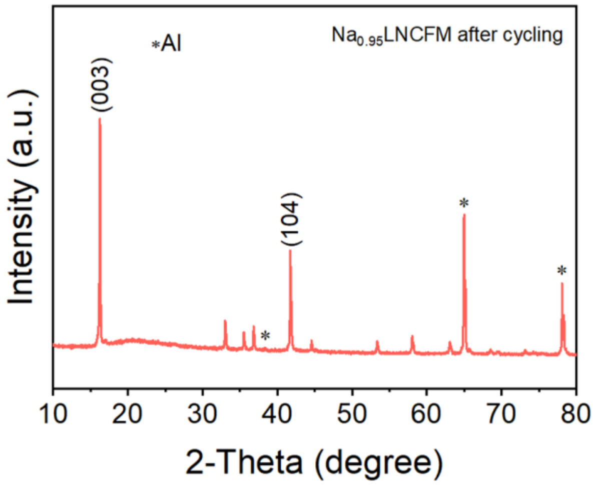

Fig. S14 XRD pattern of $Na_{0.95}Li_{0.06}Ni_{0.25}Cu_{0.05}Fe_{0.15}Mn_{0.49}O_2$ after 50 cycles

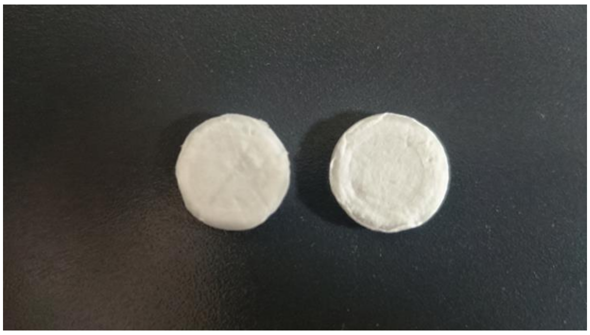

Fig. S15 Digital photographs of the separators of $Na_{0.95}$LNCFM electrode after 50 cycles

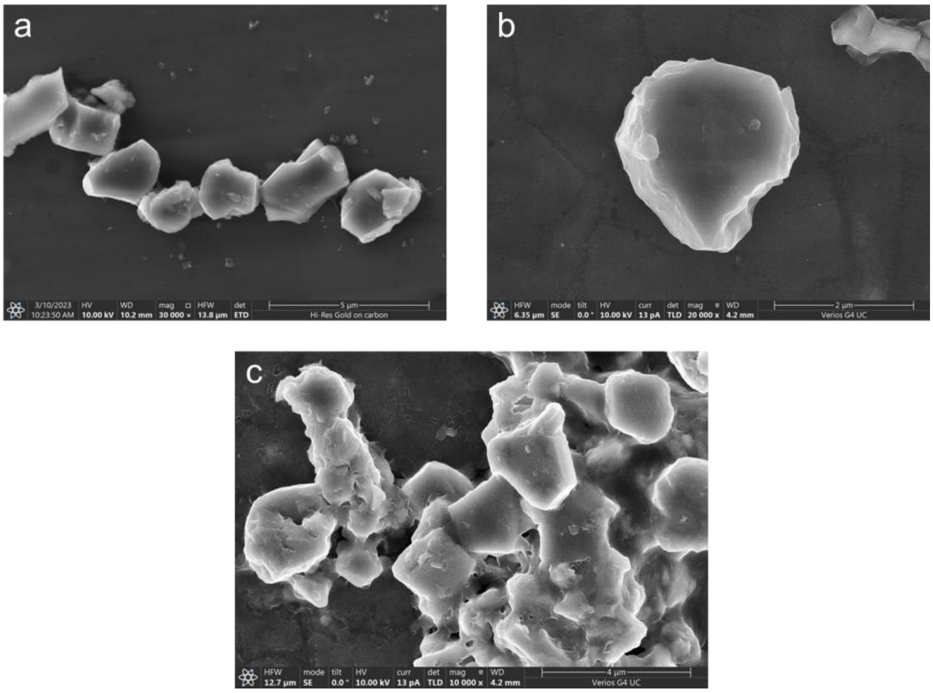

Fig. S16 SEM images of $Na_{0.95}Li_{0.06}Ni_{0.25}Cu_{0.05}Fe_{0.15}Mn_{0.49}O_2$ after 7 days exposure

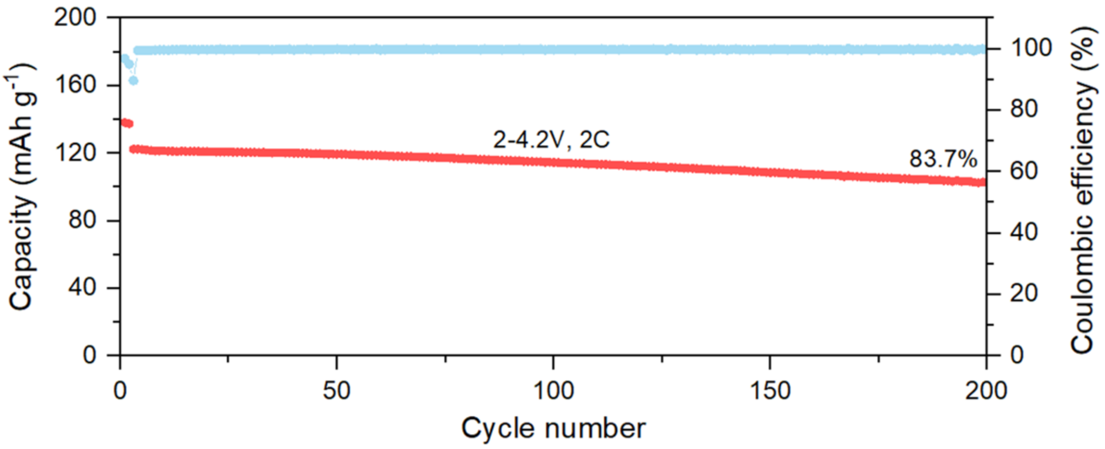

Fig. S17 Long-term cycling performance of $Na_{0.95}Li_{0.06}Ni_{0.25}Cu_{0.05}Fe_{0.15}Mn_{0.49}O_2$ after 48h exposure at 2C

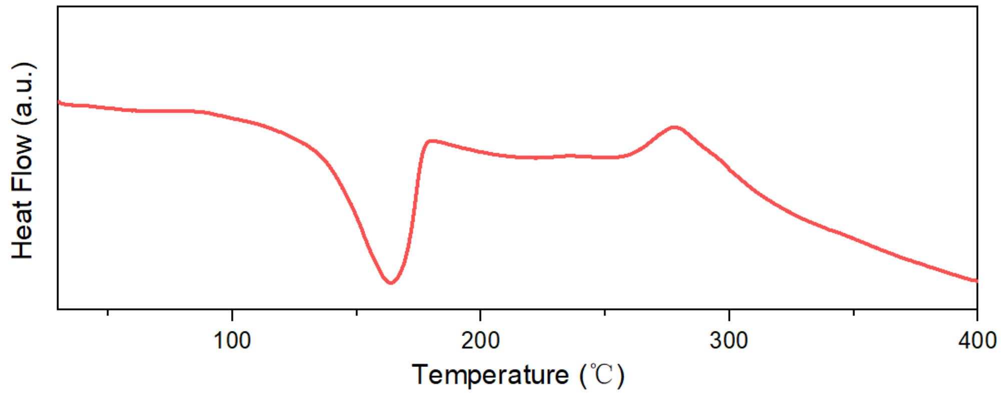

Fig. S18 The full range of the DSC curve of $Na_{0.95}$LNCFM cathode charged to 4.2 V

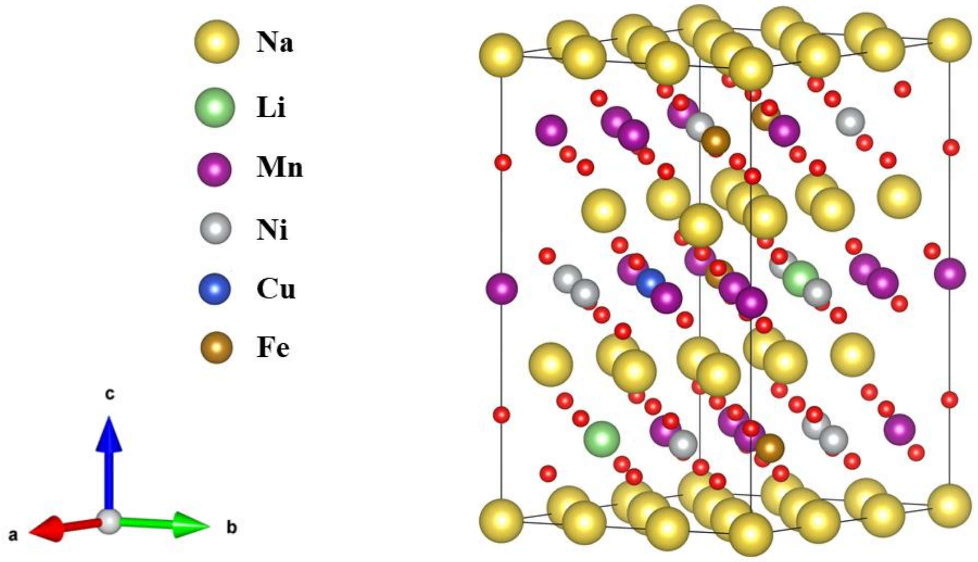

Fig. S19 Crystal structure of $Na_{0.95}$LNCFM

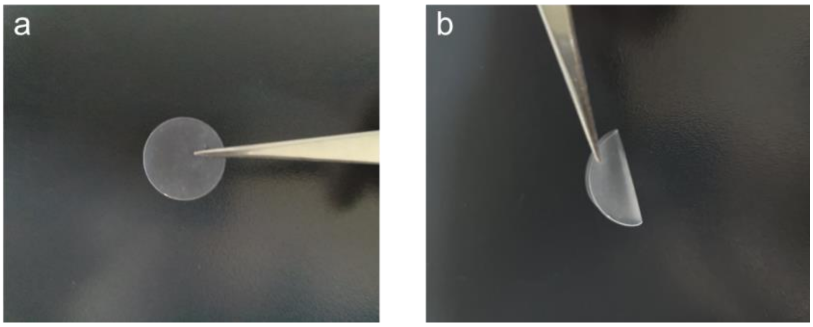

Fig. S20 Digital photographs of the PSE under different states

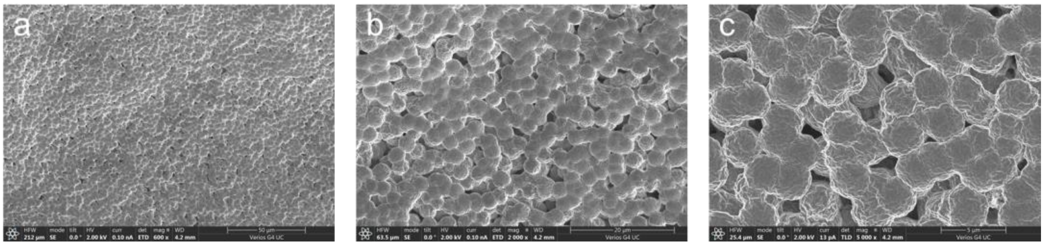

Fig. S21 SEM images of PSE

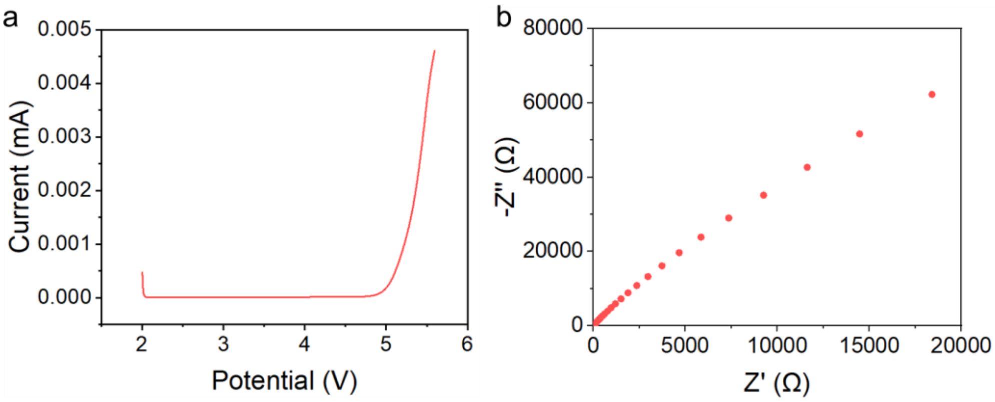

Fig. S22 a The line sweep voltammetry curve of PSE. **b** EIS spectra of PSE symmetric cell

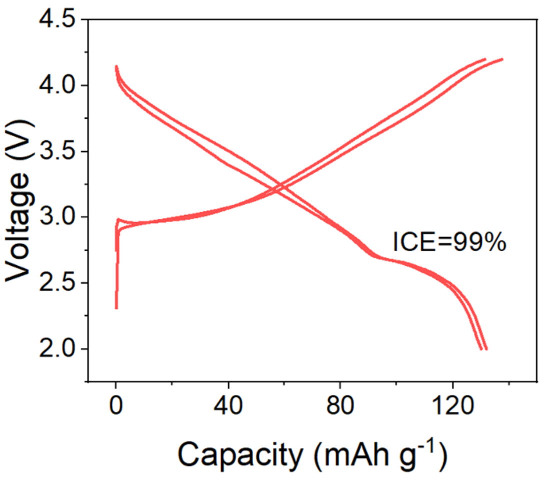

Fig. S23 The first and second charge−discharge curves at a 0.2C (1C=200 mA g^{-1}) of $Na_{0.95}$LNCFM PSE battery

Table S1 Stoichiometry of the $Na_{0.95}$LNCFM compound determined by ICP-OES

$Na_{0.95}Li_{0.06}Ni_{0.25}Cu_{0.05}Fe_{0.15}Mn_{0.49}O_2$						
mol/%	Na	Li	Ni	Cu	Fe	Mn
Measured	95.5	6.6	25.2	4.9	15	48.3
Calculated	95	6	25	5	15	49

Table S2 Specific lattice parameters of $Na_{0.95}LNCFM$ obtained by XRD Rietveld method

Atom	site	x	y	z	occ
Na	3a	0	0	0	0.95
Li	3b	0	0	0.5	0.06
Ni	3b	0	0	0.5	0.25
Fe	3b	0	0	0.5	0.15
Cu	3b	0	0	0.5	0.05
Mn	3b	0	0	0.5	0.49
O	6c	0	0	0.2324	1.00
a=b=2.94341	c=16.1641	V=121.2783	R_p:2.7%	R_{wp}=3.5%	

Table S3 Comparison of the electrochemical performance of O3-type layered cathode materials for sodium ion batteries

**Cathode materials**	**Reversible Capacity (mAh g^{-1})**	**1C rate (mA g^{-1})**	**Voltage range (V)**	**Cycling stability (capacity, retention, cycles, rate)**	**High-rate capacity (mAh g^{-1})**	**Ref**
$Na_{0.94}Ni_{0.29}Cu_{0.1}Fe_{0.16}Mn_{0.3}Ti_{0.15}O_2$	121, 0.1C	120	2.0-4.0	105, 79%, 300, 0.5C	72.5, 10C	[S1]
$Na_{0.93}Li_{0.12}Ni_{0.25}Fe_{0.15}Mn_{0.48}O_2$	130, 0.2C	200	2.0-4.2	98, 82.8%, 200, 8C	98, 8C	[S2]
$NaNi_{0.12}Cu_{0.12}Mg_{0.12}Fe_{0.15}Co_{0.15}Mn_{0.1}Ti_{0.1}Sn_{0.1}Sb_{0.04}O_2$	110.3, 0.1C	120	2.0-3.9	90, 83%, 500, 3C	108, 5C	[S3]
$Na_{0.75}Ni_{0.82}Co_{0.12}Mn_{0.06}O_2$	171, 0.1C	200	2.0-4.0	125, 65%, 400, 1C	89, 9C	[S4]
$Na(Fe_{0.2}Co_{0.2}Ni_{0.2}Ti_{0.2}Sn_{0.1}Li_{0.1})O_2$	112.7, 0.1C	100	2.0-4.1	81, 67%, 200, 0.5C	80.8. 2C	[S5]
$NaNi_{0.3}Cu_{0.1}Fe_{0.2}Mn_{0.2}Ti_{0.2}O_2$	130, 0.1C	130	2-3.9V	120, 80%, 280, 0.5C	80, 5C	[S6]
$NaFe_{0.4}Mn_{0.49}Cu_{0.1}Zr_{0.01}O_2$	147.5, 0.1C	200	2.0-4.1	140, 69%, 100, 0.2C	70.8, 5C	[S7]
$NaNi_{0.25}Mg_{0.05}Cu_{0.1}Fe_{0.2}Mn_{0.2}Ti_{0.1}Sn_{0.1}O_2$	130.8, 0.1C	140	2.0-4.0	120, 75%, 500, 1C	108, 5C	[S8]
$Na_{0.9}Cu_{0.11}Ni_{0.11}Fe_{0.30}Mn_{0.48}Ti_{0.10}O_2$	106.5, 0.1C	100	2.5-4.0	100, 89%, 500, 1C	78, 10C	[S9]
$NaNi_{0.1}Mn_{0.15}Co_{0.2}Cu_{0.1}Fe_{0.1}Li_{0.1}Ti_{0.15}Sn_{0.1}O_2$	115, 0.1C	100	2-4.1	90, 82.7%, 1000, 1.6C	90, 1.6C	[S10]
This work	141.2, 0.2C	200	2-4.2V	111, 83% .500, 8C<br>85, 85%, 1000, 20C	111, 8C<br>85, 20C	

Supplementary References

[S1] H. Guo, M. Avdeev, K. Sun, X.B. Ma, H.L. Wang et al., Pentanary transition-metals na-ion layered oxide cathode with highly reversible o3-p3 phase transition. Chem. Eng. J. **412**, 8 (2021). https://doi.org/10.1016/j.cej.2021.128704

[S2] X.G. Yuan, Y.J. Guo, L. Gan, X.A. Yang, W.H. He et al., A universal strategy toward air-stable and high-rate o3 layered oxide cathodes for na-ion batteries. Adv. Funct. Mater. **32**(17), 11 (2022). https://doi.org/10.1002/adfm.202111466

[S3] C.L. Zhao, F.X. Ding, Y.X. Lu, L.Q. Chen, Y.S. Hu, High-entropy layered oxide cathodes for sodium-ion batteries. Angew. Chem.-Int. Edit. **59**(1), 264-269 (2020). https://doi.org/10.1002/anie.201912171

[S4] J. Yang, M.J. Tang, H. Liu, X.Y. Chen, Z.W. Xu et al., O3-type layered ni-rich oxide: A high-capacity and superior-rate cathode for sodium-ion batteries. Small **15**(52), 9 (2019). https://doi.org/10.1002/smll.201905311

[S5] K.H. Tian, H. He, X. Li, D. Wang, Z.Y. Wang et al., Boosting electrochemical reaction and suppressing phase transition with a high-entropy o3-type layered oxide for sodium-ion batteries. J. Mater. Chem. A **10**(28), 14943-14953 (2022). https://doi.org/10.1039/d2ta02451a

[S6] C.C. Lin, H.Y. Liu, J.W. Kang, C.C. Yang, C.H. Li et al., In-situ x-ray studies of high-entropy layered oxide cathode for sodium-ion batteries. Energy Storage Mater. **51**, 159-171 (2022). https://doi.org/10.1016/j.ensm.2022.06.035

[S7] Y.M. Zheng, X.B. Huang, X.M. Meng, S.D. Xu, L. Chen et al., Copper and zirconium codoped o3-type sodium iron and manganese oxide as the cobalt/nickel-free high-capacity and air-stable cathode for sodium-ion batteries. ACS. Appl. Mater. Interfaces **13**(38), 45528-45537 (2021). https://doi.org/10.1021/acsami.1c12684

[S8] F.X. Ding, C.L. Zhao, D.D. Xiao, X.H. Rong, H.B. Wang et al., Using high-entropy configuration strategy to design na-ion layered oxide cathodes with superior electrochemical performance and thermal stability. J. Am. Chem. Soc. **144**(18), 8286-8295 (2022). https://doi.org/10.1021/jacs.2c02353

[S9] F.X. Ding, Q.S. Meng, P.F. Yu, H.B. Wang, Y.S. Niu et al., Additive-free self-presodiation strategy for high-performance na-ion batteries. Adv. Funct. Mater. **31**(26), 9 (2021). https://doi.org/10.1002/adfm.202101475

[S10] X.Y. Du, Y. Meng, H.Y. Yuan, D. Xiao, High-entropy substitution: A strategy for advanced sodium-ion cathodes with high structural stability and superior mechanical properties. Energy Storage Mater. **56**, 132-140 (2023). https://doi.org/10.1016/j.ensm.2023.01.010
